# Testing the price and affordability of healthy and current (unhealthy) diets and the potential impacts of policy change in Australia

**DOI:** 10.1186/s12889-016-2996-y

**Published:** 2016-04-12

**Authors:** Amanda J. Lee, Sarah Kane, Rebecca Ramsey, Elizabeth Good, Mathew Dick

**Affiliations:** School of Public Health and Social Work, Queensland University of Technology, Brisbane, QLD Australia; School of Exercise and Nutrition Sciences, Queensland University of Technology, Brisbane, QLD Australia; Preventive Health Branch, Prevention Division, Department of Health, Brisbane, QLD Australia

**Keywords:** INFORMAS, Diet prices, Food prices, Diet affordability, Food affordability, Food policy, Food environments, Healthy diets, Obesity prevention, Non-communicable disease

## Abstract

**Background:**

Price and affordability of foods are important determinants of health. Targeted food pricing policies may help improve population diets. However, methods producing comparable data to inform relevant policy decisions are lacking in Australia and globally. The objective was to develop and pilot standardised methods to assess the price, relative price and affordability of healthy (recommended) and current (unhealthy) diets and test impacts of a potential policy change.

**Methods:**

Methods followed the optimal approach proposed by INFORMAS using recent Australian dietary intake data and guidelines. Draft healthy and current (unhealthy) diet baskets were developed for five household structures. Food prices were collected in stores in a high and low SES location in Brisbane, Australia. Diet prices were calculated and compared with household incomes, and with potential changes to the Australian Taxation System. Wilcoxen-signed rank tests were used to compare differences in price.

**Results:**

The draft tools and protocols were deemed acceptable at household level, but methods could be refined. All households spend more on current (unhealthy) diets than required to purchase healthy (recommended) diets, with the majority (53–64 %) of the food budget being spent on ‘discretionary’ choices, including take-away foods and alcohol. A healthy diet presently costs between 20–31 % of disposable income of low income households, but would become unaffordable for these families under proposed changes to expand the GST to apply to all foods in Australia.

**Conclusions:**

Results confirmed that diet pricing methods providing meaningful, comparable data to inform potential fiscal and health policy actions can be developed, but draft tools should be refined. Results suggest that healthy diets can be more affordable than current (unhealthy) diets in Australia, but other factors may be as important as price in determining food choices.

**Electronic supplementary material:**

The online version of this article (doi:10.1186/s12889-016-2996-y) contains supplementary material, which is available to authorized users.

## Background

Unhealthy diets are now the major preventable risk factor contributing to the burden of disease in globally and in Australia [1] and are driven by ‘obesogenic’ food environments affecting food promotion, availability, accessibility and affordability [2]. Data from the Australian Health Survey 2011-12 [3] show that less than seven percent of Australians consume diets consistent with the recommendations of the Australian Dietary Guidelines (ADGs) 2013; [4] at least 35 % of the energy intake of adults and at least 39 % of the energy intake of children are now derived from unhealthy ‘discretionary’ choices [3], described by the Australian National Health and Medical Research Council as foods and drinks high in saturated fat, added sugar, salt and/or alcohol that are not required for health [4]. Of concern is the contribution of poor diet to the rising rates of overweight and obesity; [4] 25 % of Australian children and 63 % of Australian adults are now overweight or obese [3]. There is an urgent need for nutrition policy actions that can help shift the current intake of the whole population to a healthier diet consistent with dietary recommendations in Australia [4].

### Assessing diet price and affordability

The public perception that healthy foods are expensive is believed to be a key factor contributing to poor dietary choices and diet-related health inequities [4–6]. In developed countries, greater total spending on food tends to be associated with more nutritious dietary patterns [7]. If populations were to follow dietary recommendations, this may lead to higher food costs, with those households with the lowest incomes being most vulnerable, as they spend less per person on food, but a greater proportion of their total expenditure on food [7].

However, as the relative price of ‘healthy’ and ‘unhealthy’ foods depends on the unit of measure (i.e., per energy unit, nutrient density, serve or weight) [7], it is not always clear whether ‘healthy’ foods are really more expensive than ‘unhealthy’ foods, (e.g., healthy foods such as fruit have a high energy-to-price ratio but can provide specific nutrients at a much lower price than other less healthy foods). Comparisons can be difficult particularly in the context of the total, habitual diet that is the major determinant of diet-related disease [1, 2, 4]. Moreover, as opposed to selected pairs of ‘healthy’ and ‘less healthy’ foods, the relative price and affordability of current (unhealthy) and healthy (recommended) diets rarely have been assessed [7].

The International Network for Food and Obesity/non-communicable diseases (NCDs) Research, Monitoring and Action Support (INFORMAS), a global network of public-interest organisations and researchers that aims to monitor, benchmark and support public and private sector actions to create healthy food environments (i.e., with respect to food composition, labelling, promotion, provision, retailing, price and affordability, and trade and investment) and reduce obesity and non-communicable diseases (NCDs) and their related inequalities [2], has highlighted the need for these data [7]. INFORMAS has identified that, with respect to food price and affordability, the relevant questions which need to be answered to inform public policy are:

- What is the relative price and affordability of ‘current’ (unhealthy) and ‘healthy’ (recommended) diets?; and
- What would be the effect of potential policy actions on the relative price and affordability of ‘current’ (unhealthy) and ‘healthy’ (recommended) diets? [7]

INFORMAS proposes a step-wise framework, comprising ‘minimal’, ‘expanded’ and ‘optimal’ approaches, to monitor price and affordability of the components of ‘healthy’ and ‘less healthy’ diets, that takes into account differences in the available capacity, infrastructure and resources of countries to answer these questions and conduct monitoring activities. The framework is described in detail elsewhere [7].

In Australia, as globally, there are no standardized tools and protocols to assess and compare the price and affordability of healthy (recommended) and current (unhealthy) diets [7]. Various Australian jurisdictions and research groups apply different food baskets as dietary survey instruments, and more than ten different instruments and methods are currently in use [5, 6, 8–16]. None of these accurately reflect current Australian diets [3], nor are entirely consistent with current dietary recommendations [4, 17] (with exception of the recently revised Queensland methodology [16]). The ‘optimal’ approach of the INFORMAS diet price and affordability framework potentially provides the basis to develop a standardized method to assess and compare the price and affordability of healthy (recommended) and current (unhealthy) diets in Australia.

### Potential policy actions to improve affordability of healthy diets

A range of inter-related factors influence food prices, including political, economic, socio-cultural and environmental factors at the local, national and international levels [18, 19]. Food prices may be manipulated by governments through a range of complex policy approaches [19]. Three common pricing strategies to increase the affordability of ‘healthy’ foods at a state or national level are [7]:

(i) taxes on ‘unhealthy’ foods e.g., on sugar-sweetened beverages;
(ii) exemption of ‘healthy’ foods from goods and service tax (GST) or value added tax; and
(iii) subsidies on ‘healthy’ foods such as agricultural and transport subsidies, retail price reductions, or voucher systems targeted to high-risk groups.

In Australia, basic healthy foods (such as fruit, vegetables, bread, fresh meat, milk and eggs) are currently exempt from GST, which is applied at a rate of 10 % to all other foods and drinks [20]. The GST status of specific food items in Australia can be determined online at the Australian Taxation Office calculator website [21]. Despite basic healthy foods being exempt from GST in Australia, the limited available (non-comparable) data suggest that the cost of ‘healthy’ food is higher [5] and has increased more rapidly than the cost of ‘unhealthy’ food over the last 15 years [14, 16]. Similar findings have been described internationally [7, 22].

As a revenue raising measure, the Australian Government has proposed expanding the GST base by removing exemption of basic healthy foods and/or increasing the rate of GST above 10 % [20]. While the potential effects of these proposed changes on the price of specific, selected foods have been modelled [23, 24], the potential impact on price and affordability of total current and recommended diets has not been determined.

### Aim

This study pilots the development and testing of draft tools and protocols with the potential to be standardized nationally, consistent with the INFORMAS ‘optimal’ approach [7], to investigate the price, relative price and affordability of healthy (recommended) and current (unhealthy) diets in Australia. It also tests the utility of these methods in determining the effect of potential fiscal policy actions by investigating, as an example, the impacts of the mooted broadening and/or raising of the GST base in Australia on the relative price and affordability of current (unhealthy) and healthy (recommended) diets.

## Methods

### Construction of the draft diet basket pricing tools

Diet baskets were developed for five different household types and structures, in expansion of the one reference household proposed by the INFORMAS approach, to encompass the major options used previously in food price surveys in Australian states and territories and optimize utility: [6, 9, 12, 14, 16]

- Household 1 (HH1) (*n* = 6): adult male 31–50 year old; adult female 31–50 year old; older female 70+ yrs old; boy 14 years old; girl 8 years old; boy 4 years old
- Household 2 (HH2) (*n* = 3): single parent with 2 children: adult female 31–50 year old; boy 14 years old; girl 8 years old
- Household 3 (HH3) (*n* = 1): single unemployed person: adult male 31–50 year old
- Household 4 (HH4) (*n* = 2): older couple with no children: senior adult male 70+ yrs old; senior adult female 70+ yrs old: pensioners
- Household 5 (HH5) (*n* = 4): adult male 31–50 year old; adult female 31–50 year old; boy 14 years old; girl 8 years old

Development of the current (unhealthy) diet basket followed the INFORMAS optimal approach [7]. It contains foods and quantities based on the results of the dietary component of the Australian Health Survey (AHS) 2011–12 which report major, sub-major and minor food groups by age/gender groups (ABS: data cubes 5.1 and 5.3) [3]. When the draft tool was developed in August 2014, confidential unit record files (CURFs) from the survey had not been released, but it was anticipated that application of the summary data available publically could inform a relatively pragmatic method to estimate current diets, suitable for broader global application under INFORMAS [7]. The current (unhealthy) diet basket is comprised of foods and drinks in the quantities and proportions reported as consumed, for example, with percentage energy from discretionary choices as reported earlier [3] (See Additional file 1: file 2.1).

Ideally, the specific foods included in both diet baskets are culturally acceptable, commonly consumed, widely available, accessible and considered ‘every day’ rather than luxury items. As the foods and drinks included in the current (‘unhealthy’) basket reflect actual consumption data, it was presumed that they were deemed by the population as a whole as meeting these requirements.

For the current (unhealthy) diet baskets, the mean amounts of each food sub-group per fortnight were calculated from reported daily intake for each relevant age/gender group (ABS: Table 5.1 and Table 5.3) [3] and tallied.

Development of the healthy (recommended) diet basket also followed the INFORMAS optimal approach [7] based on the quantitative modelled Foundation Diet recommendations for each age/gender group of the Australian Guide to Healthy Eating which include modelled serve sizes and recommended number of serves [4, 17]. The Foundation diet serve recommendations for household members are shown in Table 1. The products in the healthy diet basket are analogous to the commonly consumed healthy foods in the current diet, but differ in quantity. The healthy diet basket for each household contains the recommended quantities of foods from the five food groups and an allowance for unsaturated oils and spreads, for composite age/gender groups of physical activity level (PAL) 1.4, but, consistent with Australian recommendations [4, 17],does not contain any discretionary choices [4]. As the Foundation Diets were developed for the smallest adults (or in the case of children, the youngest) in each age/gender group, any necessary adjustments were made for height/age; for example, amounts were adjusted by an additional 20 % for the 8 year old girl who was the oldest in her group [17]. Consistent with the modelling [17], the healthy diet basket includes: grain (cereal) foods, in the ratio 2/3 wholegrain and 1/3 refined varieties; cheese, milk, yoghurt and calcium-fortified plant based alternatives, mostly (i.e., >50 %) reduced fat, with a maximum of 2–3 serves of high fat dairy foods (cheese) per week; lean meat (beef, lamb, veal, pork), poultry and plant-based alternatives (with no more than 455 g red meat per week); a minimum of 140 g and up to 280 g fish per week; around 7 eggs per week; a selection of different colours and varieties of vegetables (green and brassica, orange, legumes, starchy veg, other) with a minimum 350 g per day for adults; a variety of fruit with a minimum of 300 g per day for adults; and an allowance of unsaturated oils or spreads or the nuts/seeds from which they are derived. (See Additional file 1: file 1.1).

**Table 1:** Foundation diets: recommended numbers of serves per week to comprise the healthy diet baskets

		Omnivore Foundation Diets (modelled serves per week)												
		Starchy veges	Green & brassica veges	Orange veges	Legumes	Nuts & seeds	Other veges	Fruit	Wholegrain or higher fibre cereals/grains	Refined or lower fibre cereals/grains	Meats & alts (minus red)	Red meats	Diary foods 50 % reduced fat	Poly-unsaturated margarine^a^
Household structure	Members	75 g	75 g	75 g	75 g	30 g	75 g	150 g	Equiv 40 g bread	Equiv 40 g bread	Equiv 65 g meats	65 g	Equiv 250g milk	10 g
1	Female 70+ yrs	5	7	7	3	3	14	14	20	8	7	3	28	14
	Male 31–50 year	7	7	7	7	7	14	14	28	14	7	7	17	28
	Female 31–50 year	5	7	7	2	2	14	14	28	14	7	7	17	14
	Male 14 years	7	7	7	2	4	14	14	32	17	7	7	25	14
	Female 8 years	3.5	7	7	2	0	10.5	10.5	19	9	5.5	5	11.5	5
	Male 4 years	3.5	7	7	2	0	10.5	10.5	19	9	5.5	5	14	5
	TOTAL	31	42	42	18	16	77	77	146	71	39	34	112.5	80
2	Female 31–50 year	5	7	7	2	2	14	14	28	14	7	7	17	14
	Male 14 years	7	7	7	2	4	14	14	32	17	7	7	25	14
	Female 8 years	3.5	7	7	2	0	10.5	10.5	19	9	5.55	5	11.5	5
	TOTAL	15.5	21	21	6	6	38.5	38.5	79	40	19.5	19	53.5	33
3	Male 31–50 year	7	7	7	7	7	14	14	28	14	7	7	17	28
	TOTAL	7	7	7	7	7	14	14	28	14	7	7	17	28
4	Female 70 + yrs	5	7	7	3	3	14	14	20	8	7	3	28	14
	Male 70 + yrs	7	7	7	2	4	14	14	28	14	7	7	17	28
	TOTAL	12	14	14	5	7	28	28	48	22	14	10	45	42
5	Male 31–50 year	7	7	7	7	7	14	14	28	14	7	7	17	28
	Female 31–50 year	5	7	7	2	2	14	14	28	14	7	7	17	14
	Male 14 years	7	7	7	2	4	14	14	32	17	7	7	25	14
	Female 8 years	3.5	7	7	2	0	10.5	10.5	19	9	5.5	5	11.5	5
	TOTAL	22.5	28	28	13	13	52.5	52.5	107	54	26.5	26	70.5	61

Each household allocated 100 g polyunstructured oil and the rest of the allowance provided as polyunstructured margarine

Serve sizes as specefied in the Australian Dietary Guidelines Educators’Guide at https://www.eatforhealth.gov.au/sites/default/files/files/the_guidelines/n55b_educator_guide_140321.pdf

For the healthy (recommended) diet baskets, the amounts of each food sub-group (serve size multiplied by recommended number of serves) per fortnight were calculated from omnivore Foundation Diets [17] for each of the specific age/gender groups included in the five household structures and tallied. A variety of fresh, canned, frozen and dried foods was included in the proportions modelled [17] with representative fresh produce being in season for most of the year (e.g., stone fruits in season only in summer were excluded). Luxury products such as imported fruit and vegetables (particularly out of season) and foods with high cost per kilogram (e.g., oysters, smoked salmon) were excluded.

The specific branding of items and product sizes for pricing were not finalized until field testing confirmed that these products were commonly available in pilot stores (See Additional file 1: files 1.2 and 2.2).

### Collection of food prices

One high socio-economic and one low socio-economic Statistical Area Level 2 (SA2) [25] location within 30 km of Brisbane General Post Office were randomly selected as locations for field testing; these were Indooroopilly (high SES) and Logan (low SES). In both locations, stores of the national supermarket chains available in all state and territories in Australia (Coles, Woolworths and Independent Grocers Australia (IGA)) were included, and the prices of food and drink items in both diet baskets were collected in the second and third weeks of September 2014, including commonly available brands, sizes and ‘home-brand’ generic items, based on methods previously used in Western Australia [14]. Common ‘fast-food’/take-away items (including a Big Mac hamburger from the McDonald’s™ chain, pizza from the Pizza Hut™ chain and fish and chips from independent outlets) and alcoholic beverages (spirits, wine and beer) were priced in the respective take-away and liquor outlets closest to each supermarket in each location.

Data recorded included: usual price as well as the price promotion or sale price; cheapest price for loose (not packaged) fresh produce of that description (e.g., red apples); brand name, and whether the item is branded or generic (‘home brand’); and prices of different available sizes.

To finalise the diet baskets, the most commonly available branded items and unit sizes in all six supermarkets were included. Some nutritionally similar products were combined to minimize the number of items in each tool; for example, processed meats such as salami and hot dogs were combined with sausages (See Additional file 1: files 1.2 and 2.2).

No attempt was made to adjust the quantities in the current (unhealthy) diet basket for under-reporting in the AHS 2011–12 [3]. An allowance for edible portion/as cooked was included in both diet baskets, but not for any post-plate wastage in either basket (Additional file 1: files 1.2 and 2.2).

### Performance of draft tools and protocols

Convergent validity of the constructed healthy (recommended) and current (unhealthy) diet baskets for each age/gender group was assessed by energy and macronutrient analysis using FoodWorks 7 Professional [26] computer program installed with current Australian food composition datasets, and comparing the results with Australian Nutrient Reference Values [27] and nutrient results from the AHS 2011-12 [3] respectively. The food composition database used to analyse the AHS (AUSNUT 2011–13) was not available publically at the time this manuscript was prepared; comparative analysis could be improved by repeating analysis when it is released publically. Price and affordability findings were compared with previous available estimates to determine face validity. Internal validity indicators, such as the ratio of fruit and vegetables between diet baskets, were also assessed and compared.

### Diet prices and affordability

The price of the final ‘healthy’ (recommended) and current (unhealthy) diet baskets was calculated for each of the five household compositions in each store in each location.

The potential price increases for all foods in the diet baskets under the mooted changes to the Australian tax system [20] were also determined by application of the information available at the Australian Taxation Office calculator site for businesses [21]. Changes in price were compared to identify potential impacts on diet cost for each household (See Additional file 1: files 1.3, 1.4, 1.5, 2.3, 2.4, 2.5).

Affordability of the healthy (recommended) and current (unhealthy) diet baskets for the average family household was determined by comparing the cost of each diet with the median disposable household income, as per the INFORMAS optimal approach [7]. Median disposable household income derived from recent census data in both locations was transcribed from the Queensland Government Statistician’s Office website [28].

For the purposes of calculating the affordability of the healthy (recommended) and current (unhealthy) diet baskets for low income families, indicative minimum disposable income of each household was calculated based on the level of minimum wages and determination of the welfare payments provided by the Department of Human Services (2014) as per the methods used by the Queensland Department of Health [16]. Based on the Queensland Council of Social Services’ cost of living research on theoretical low income households [29] assumptions were made for employment, housing type, disability status, savings and investments, child support, education attendance and immunization status of children [16]. Details are included in Table 2.

**Table 2:** Household structures and associated income

	Household 1: Household of 6	Household 2: Single parent with 2 children	Household 3: Single unemployed person	Household 4: Older couple with no children	Household 5: Two parents with two children
	Adult male, adult female, older female, 14 years boy, 8 years girl, 4 years boy	Adult female, 14 years boy, 8 years girl	Adult male	Older male, older female	Adult male, adult female, 14 years boy, 8 years girl
Assumptions	• The adult male and female are partnered parents of the three dependent children • Adult male is unemployed and looking for work • Adult female is a stay-at-home mum • Older female receives age pension • The older children attend school and are fully immunised • The youngest child attends kindy 2 days/week and is fully immunised • The family does not have savings or investments • The family is living in public housing	• The adult female works on a casual basis at national minimum wage ($20.30/h) for 25 h a week for 39 weeks per year (not during school holidays) • The adult female does not receive child support from the children’s father • Both children attend school and are fully immunised • None of the family are disabled • The family does not have savings or investments • The family is privately renting their home at $332/week	• Has no paid employment but is looking for work • Is not studying/training • Is not disabled • Has no dependent children • Does not have savings or investments • Is renting a room in 3 bedroom house at $122/week	• Neither are in paid employment • Both receive the full age pension (maximum rate) • Neither are disabled or frail-aged • The couple has no dependent children • The couple has some savings earning $100/fortnight in ‘deemed income’ (less than asset test for age pension and as in a term deposit and not easily accessed, not included in fortnightly income) • The couple is privately renting their home at $322/week	• The adult male works on a permanent basis at national minimum wage for 38 h a week ($16.37/h) • The adult female works on a partime basis at national minimum wage ($16.37/h) for 6 h a week • Both children attend school and are fully immunised • None of the family are disabled • The family has some emergency savings that earn negligible interest • The family is privately renting a 3 bedroom house at $365/week
INCOME per fortnight					
Paid employment	Nil	$761.25 (average over year)	Nil	Nil	$1244.12 (adult male) $196.44 (adult female)
Newstart Allowance	$460.90 (adult male)	N/A	$510.50 (receives maximum)	N/A	N/A
Parenting Payment	$460.90 (adult female)	N/A (as youngest child is not under 8 years)	N/A	N/A	N/A (as youngest child is not under 8 years)
Family Tax Benefit A and supplement	$568.40/fortnight and $2,179.05/year ($83.80/fortnight)	$396.20/fortnight and $1,452.70/year ($55.87/fortnight)	N/A	N/A	$396.20/fortnight and $1,452.70/year ($55.87/fortnight)
Family Tax Benefit B and supplement	$146.44/fortnight and $354.04/year ($13.61/fortnight)	$102.20/fortnight and $354.04/year ($13.61/fortnight)	N/A	N/A	$102.20/fortnight and $354.04/year ($13.61/fortnight)
Age Pension	$766.00 (older female)	N/A	N/A	$1,154.80 (couple payment)	N/A
Age Pension Supplement	$62.90 (older female)	N/A	N/A	$94.80	N/A
Clean Energy Supplement	$43.26	$9.69	$8.70	$21.00	$9.69
Rent Assistance	N/A (as live in public housing)	$147.98	$84.27	$118.80	$147.98
Income Support Bonus	$359.60/year ($13.83/fortnight) (adult male and female)	N/A	$215.60/year ($8.29/fortnight)	N/A	N/A
Low Income Supplement	N/A	N/A	N/A	N/A	N/A
Low Income Family Supplement	N/A	$300/year ($11.53/fortnight)	N/A	N/A	$300/year ($11.53/fortnight)
Single Income Family Supplement	N/A	N/A	N/A	N/A	N/A
Large Family Supplement	$12.04	N/A	N/A	N/A	N/A
School Kid Bonus	$1,230/year ($47.30/fortnight)	$1,230/year ($47.30/fortnight)	N/A	N/A	$1,230/year ($47.30/fortnight)
Childcare benefit	Paid directly to kindergarten	N/A	N/A	N/A	N/A
Childcare rebate	Paid directly to kindergarten	N/A	N/A	N/A	N/A
INCOME TAX PAID	Nil	Nil due to low income tax offset ($302 withheld from wages is refunded with tax return)	Nil	Nil	$2,278/year ($87.62/fortnight) (after allowing for low income tax offset)
TOTAL	$2,679.38/fortnight	$1,545.63/fortnight	$611.76/fortnight	$1,389.40/fortnight	$2,137.32/fortnight

Source: Based on Queensland Department of Health 2015 (16)

1) Government payments based on payments and rates available April 2014 at: www.humanservices.gov.au

2) Salaries calculated on national minimum wage at www.fairwork.gov.au $17.29/h, casual add 25 % loading to $21.61/h

3) Work status based on 2011 Census data for Queensland (couple households tend to have a fulltime working male and a part-time working female; or when the male is looking for work the female is typically also out-of-work; for single-parent households, more females tend to work part-time until children are over 14 years)

4) Average rent varies by location (SA2): http://www.abs.gov.au/ausstats/abs@.nsf/Lookup/by%20Subject/4130.0~2013–14~Main%20Features~Housing%20Costs%20and%20Affordability~5 Estimated rent based on average of median rates across Queensland postcodes for March 2014 quarter from the Rental Tenancy Authority

5) As all households exceeded maximum fortnightly rent, maximum rent assistance was paid

6) Immunisation status and child support assumed to be able to receive the maximum Government payments

### Data and statistical analysis

Results were analyzed by a range of metrics, including the current cost of purchasing specific core five food group and discretionary items (including policy relevant items such as alcohol, ‘take-away foods’ and sugar-sweetened beverages) and compared with the component costs of healthy (recommended) diets.

Data were also entered into IBM SPSS statistics Version 21 and Wilcoxen-signed ranks test used to compare the costs of diets between high and low socioeconomic (SES) areas and differences in the cost of diets based on proposed policy changes to GST.

## Results

### The diet basket pricing tools

The daily nutrient analysis of the healthy (recommended) and current (unhealthy) diets for each age/gender group included in the five household structures is presented in Table 3. The foods comprising the diet baskets are presented in Table 4.

**Table 3:** Energy and macronutrient analysis of constructed healthy (recommended) [3] and current (unhealthy) diets [4] for select age/gender groups used to develop the household diet baskets

Age and gender group	Diet	Dietary Analysis					
		Energy intake (kJ/day)	% Energy from protein	% Energy from total fat	% Energy from saturated fat	% Energy from carbohyd-rate	% Energy from alcohol
Adult male 19–50 year	Constructed current (unhealthy) diet^a^	10670	17	32	12	47	4
	Mean reported intake (AHS) [1]	10220	18	32	12	43	6
	Constructed healthy diet^a^	8750	22	33	9	45	0
	Recommended Foundation diet (Ht 1.6 m PAL 1.4) [2]	9000	15-25	20-35	<10	45-65	<5
Adult female 19–50 year	Constructed unhealthy (current) diet^a^	7820	17	30	12	49	4
	Mean reported intake (AHS) [1]	7540	18	33	12	44	4
	Constructed healthy diet^a^	7370	24	29	9	47	0
	Recommended Foundation diet (Ht 1.5 m PAL 1.4) [2]	7100	15-25	20-35	<10	45-65	<5
Senior male ≥ 70 year	Constructed current (unhealthy) diet^a^	9040	16	30	12	48	4
	Mean reported intake (AHS) [1]	8170	17	31	12	45	5
	Constructed healthy diet [2]	7460	26	30	11	43	0
	Recommended Foundation diet (Ht 1.6 m PAL 1.4) [2]	7300	15-25	20U-35	<10	45-65	<5
Senior female ≥ 70 year	Constructed current (unhealthy) diet^a^	7200	16	30	12	50	3
	Mean reported intake (AHS) [1]	6570	18	32	12	44	4
	Constructed healthy diet^a^	6710	25	32	12	44	0
	Recommended Foundation diet (Ht 1.5 m PAL 1.4) [2]	6500	15-25	20-35	<10	45-65	<5
Boy 14 years	Constructed current (unhealthy) diet^a^	9130	16	29	12	54	0
	Mean reported intake (AHS) [1]	10190	17	33	13	49	0
	Constructed healthy diet^a^	8770	23	28	9	47	0
	Recommended Foundation diet (PAL 1.4) [2]	9300	15-25	20-35	<10	45-65	0
Girl 8 years	Constructed current (unhealthy) diet^a^	6420	15	27	12	56	0
	Mean reported intake (AHS) [1]	6430	15	31	13	52	0
	Constructed healthy diet [3]^a^	5890	26	23	9	51	0
	Recommended Foundation diet (PAL 1.4) [2]	6000	15-25	20-35	<10	45-65	0
Boy 4 years	Constructed current (unhealthy) diet^a^	7650	15	30	12	54	0
	Mean reported intake (AHS) [1]	7640	15	32	14	51	0
	Constructed healthy diet^a^	5110	25	24	10	50	0
	Recommended Foundation diet (PAL 1.4) [2]	5200	15-25	20-35	<10	45-65	0

^a^Analysed in FoodWorks™ 7 Professional using NUTTAB 2010 and AusBrands 2012 data bases

**Table 4:** Foods comprising healthy (recommended) & current (unhealthy) diet baskets, five household (HH) structures

Current (unhealthy) diet basket					
	HH 1^a^	HH 2^b^	HH 3^c^	HH 4^d^	HH 5^e^
Constructed diets: Total energy per HH per day (kJ)	48890	23370	10670	16240	34040
Reported diets: Total energy per HH per day (kJ)	48590	24160	10220	14740	34380
Food	Total amount per fortnight				
Fruit					
Apples, loose (g)	3100	1700	500	1100	2200
Bananas, loose (g)	3200	1700	500	1200	2200
Oranges, loose (g)	3200	1700	500	1200	2200
Fruit salad, canned in juice (g)	3100	1700	500	1100	2200
Vegetables					
Potato, loose (g)	2100	1050	400	800	1450
Sweetcorn, canned (g)	510	240	100	200	340
Broccoli, loose (g)	300	130	70	140	200
White cabbage, loose (g)	300	130	70	140	200
Iceberg lettuce, whole (g)	540	260	100	200	360
Carrot, loose (g)	760	340	160	320	500
Pumpkin (g)	1100	500	200	500	700
Four bean mix, canned (g)	750	350	150	300	500
Diced tomatoes, canned (g)	1600	650	400	800	1050
Onion, loose (g)	540	260	100	200	360
Tomatoes, loose (g)	900	400	200	400	600
Frozen mixed vegetables, pre-packaged (g)	900	400	200	400	600
Frozen peas, pre-packaged (g)	700	300	200	300	500
Baked beans, canned (g)	825	475	125	250	600
Grain (cereal) foods					
Weetbix	1400	700	250	450	950
Wholemeal bread, pre-packaged (g)	700	350	150	250	500
Rolled oats (g)	600	300	100	200	400
White bread, pre-packaged (g)	4600	2200	950	1550	3150
Cornflakes (g)	1400	700	250	450	950
White pasta (g)	1650	800	300	550	1100
White rice (g)	1650	800	300	550	1100
Meats, poultry, fish, eggs, nuts and seeds					
Beef mince (g)	800	375	200	300	575
Lamb loin chops (g)	790	370	200	300	570
Beef rump steak (g)	840	420	200	300	620
Tuna, canned in springwater (g)	1145	500	285	560	785
Chicken breast (g)	1750	730	460	640	1190
Eggs (g)	1140	565	240	460	805
Canned meat and vegetable casserole (g)	2350	1200	600	650	1800
Milk, yoghurt, cheese and alternatives					
Cheddar cheese, full fat (g)	720	370	120	200	490
Milk, full fat (ml)	9850	4850	1600	2900	6450
Cheddar cheese, reduced fat (g)	310	160	55	80	215
Milk, reduced fat (ml)	6350	3150	1000	1900	4150
Yoghurt, reduced fat (g)	910	440	130	220	570
Yoghurt, flavoured reduced fat (g)	150	0	150	100	150
Unsaturated oils and spreads					
Canola margarine (g)	120	50	25	65	75
Sunflower oil (ml)	120	50	25	65	75
Discretionary choices					
Beer, full strength (ml)	7235	2639	3569	2986	6208
White wine, sparkling (ml)	1454	482	498	1014	980
Whisky (ml)	163	51	71	107	122
Red wine (ml)	1454	482	498	1014	980
Butter (g)	364	181	58	163	239
Muffin, commercial (g)	3485	1521	747	1217	2268
Cream-filled sweet biscuit, pre-packaged (g)	933	320	332	405	652
Muesli bar, pre-packaged (g)	528	322	83	81	405
Peanuts, salted (g)	295	108	83	137	191
Pizza, commercial (g)	3013	1610	830	573	2440
Savoury flavoured biscuits (g)	1298	642	249	407	891
Confectionary (g)	662	342	116	146	458
Chocolate (g)	321	169	58	61	227
Coca Cola (ml)	14117	7468	4026	2284	11493
Meat pie, commercial (g)	1209	632	332	245	964
Frozen lasagne, pre-packaged (g)	1381	767	332	490	1099
Hamburger, commercial (g)	1306	609	332	324	941
Beef sausages (g)	1557	486	664	822	1150
Ham (g)	1059	403	332	490	735
Potato crisps, pre-packaged (g)	673	367	71	44	438
Potato chips, commercial (g)	1176	565	249	486	814
Ice cream (g)	1948	806	332	727	1138
White sugar (g)	1492	724	208	528	931
Salad dressing (ml)	616	305	133	153	437
Tomato sauce (ml)	641	305	133	153	437
Chicken soup, canned (g)	2714	1078	598	1753	1675
Orange fruit drink (ml)	8201	4485	1245	1861	5730
Fish fillet crumbed, pre-packaged (g)	535	266	108	211	374
Healthy (recommended) diet basket					
	HH 1^a^	HH 2^b^	HH 3^c^	HH 4^d^	HH 5^e^
Constructed diets: Total energy per HH per day (kJ)	42600	22030	8750	14170	30780
Recommended diets: Total energy per HH per day (kJ)	43100	22400	9000	13800	31400
Food	Total amount per fortnight				
Fruit					
Apples, loose (g)	7910	4060	1400	2800	5460
Bananas, loose (g)	7910	4060	1400	2800	5460
Oranges, loose (g)	7910	4060	1400	2800	5460
Vegetables					
Potato, loose (g)	2970	1620	700	800	2320
Sweetcorn, canned (g)	1485	810	350	400	1160
Broccoli, loose (g)	2170	1120	350	700	1470
White cabbage, loose (g)	2170	1120	350	700	1470
Iceberg lettuce, whole (g)	2170	1120	350	700	1470
Carrot, loose (g)	3255	1680	525	1050	2205
Pumpkin (g)	3255	1680	525	1050	2205
Four bean mix, canned (g)	2760	960	1050	750	2010
Diced tomatoes, canned (g)	2373	1218	420	840	1638
Onion, loose (g)	2373	1218	420	840	1638
Tomatoes, loose (g)	2373	1218	420	840	1638
Frozen mixed vegetables, pre-packaged (g)	2373	1218	420	840	1638
Frozen peas, pre-packaged (g)	2373	1218	420	840	1638
Grain (cereal) foods					
Weetbix	2896	1656	560	720	2216
Wholemeal bread, pre-packaged (g)	5792	3312	1120	1440	4432
Rolled oats (g)	8688	4968	1680	2160	6648
White bread, pre-packaged (g)	1133	669	224	256	893
Cornflakes (g)	850	502	168	192	670
White pasta (g)	2124	1254	420	480	1674
White rice (g)	2124	1254	420	480	1674
Wholegrain crackers (g)	991	585	196	224	781
Meats, poultry, fish, eggs, nuts and seeds					
Beef mince (g)	1514	865	303	433	1168
Lamb loin chops (g)	1516	866	303	433	1169
Beef rump steak (g)	1518	867	304	434	1171
Tuna, canned in springwater (g)	2675	1374	467	934	1841
Chicken breast (g)	2137	1098	373	746	1471
Eggs (g)	3208	1648	560	1120	2208
Peanuts, unsalted (g)	960	360	420	420	780
Milk, yoghurt, cheese and alternatives					
Cheddar cheese, full fat (g)	1104	544	160	520	704
Milk, full fat (ml)	7651	3713	1125	3375	4838
Cheddar cheese, reduced fat (g)	408	198	60	180	258
Milk, reduced fat (ml)	26867	13034	4000	12167	17034
Yoghurt, reduced fat (g)	10746	5213	1600	4867	6813
Unsaturated oils and spreads					
Canola margarine (g)	538	226	186	186	412
Sunflower oil (ml)	757	318	261	262	579

*HH* Household

^a^HH1 (*n* = 6): male 19–50 year; female 19–50 year; female 70 + yrs; boy 14 years; girl 8 years; boy 4 years

^b^HH2 (*n* = 3): female 19–50 year; boy 14 years; girl 8 years

^c^HH3 (*n* = 1): male 19–50 year

^d^HH4 (*n* = 2): male 70+ yrs; female 70 + yrs

^e^HH5 (*n* = 4): adult male 19–50 year old; adult female 19–50 year old; boy 14 years old; girl 8 years

As deemed acceptable for modelling outputs to develop the Australian Guide to Healthy Eating [17], the energy content of the constructed healthy (recommended) diet baskets was within 5 % of the Foundation Diet levels and the macronutrient profiles were within the recommended ranges for more than 97 % of values for all age/gender groups [17]. Hence these tools appeared to be valid for use in estimating the cost of healthy diets in this pilot. The energy content of the current (unhealthy) diet baskets was within 5 % of the reported energy intakes of the AHS [3] for the adult male, adult female and the two youngest children, but not for the 14 year old boy or seniors (Table 3). However, at the household level, the mean energy content of the current (unhealthy) diet basket was within 5 % of the reported intakes for all but HH4 (comprising the two seniors) (Table 4). However, as the price findings (Table 5) were consistent with available household expenditure survey data [30] (discussed below) and reflected the ratio of current and recommended intakes for key food groups [3, 4], the current (unhealthy) diet basket tools appeared to be valid for use in estimating the cost of current diets in the majority of the households in this pilot study. However results for HH4 should be applied with caution.

**Table 5:** Price of healthy (recommended) and current (unhealthy) diet baskets for five household structures at two locations in Queensland

Location	Logan										Indooroopilly									
Diet Basket Components	HH 1 Healthy diet	HH 1 Current (unhealthy) diet	HH 2 Healthy Diet	HH 2 Current (unhealthy) diet	HH 3 Healthy Diet	HH 3 Current (unhealthy) diet	HH 4 Healthy Diet	HH 4^a^ Current (unhealthy) diet	HH 5 Healthy diet	HH 5 Current (unhealthy) diet	HH 1 Healthy diet	HH 1 Current (unhealthy) diet	HH 2 Healthy Diet	HH 2 Current (unhealthy) diet	HH 3 Healthy Diet	HH 3 Current (unhealthy) diet	HH 4 Healthy Diet	HH 4^a^ Current (unhealthy) diet	HH 5 Healthy diet	HH 5 Current (unhealthy) diet
Total diet basket $mean ± sd	795.47 ± 25.76	896.04 ± 5.28	414.50 ± 13.66	418.71 ± 1.59	146.43 ± 4.53	221.83 ± 1.22	272.88 ± 8.67	325.99 ± 2.42	560.93 ± 18.19	640.20 ± 2.67	822.85 ± 55.61	925.69 ± 45.53	428.84 ± 28.77	432.75 ± 20.16	151.17 ± 9.89	229.44 ± 9.84	282.17 ± 19.43	336.68 ± 15.67	580.01 ± 38.66	661.92 ± 30.00
Fruit and Vegetables $mean ± sd (% total basket price)	239.43 ± 19.11 (30.10 %)	120.22 ± 6.89 (13.42 %)	121.61 ± 9.82 (29.34 %)	61.30 ± 3.69 (14.64 %)	44.95 ± 3.38 (30.70 %)	21.62 ± 1.14 (9.75 %)	80.91 ± 6.72 (29.65 %)	46.38 ± 2.54 (14.23 %)	166.56 ± 13.20 (29.69 %)	82.92 ± 4.83 (12.95 %)	254.02 ± 28.13 (30.87 %)	124.12 ± 10.51 (13.41 %)	128.99 ± 14.32 (30.08 %)	63.35 ± 5.53 (14.64 %)	47.53 ± 4.98 (31.44 %)	22.33 ± 1.87 (9.73 %)	86.06 ± 9.79 (30.50 %)	47.82 ± 3.94 (14.20 %)	176.53 ± 19.30 (30.44 %)	85.68 ± 7.40 (12.94 %)
Core five food group foods $mean ± sd (% total Basket price)	795.47 ± 25.76 (100.0 %)	386.95 ± 11.95 (43.19 %)	414.50 ± 13.66 (100.0 %)	188.20 ± 5.88 (44.95 %)	146.43 ± 4.53 (100.0 %)	80.25 ± 2.55 (36.18 %)	272.88 ± 8.67 (100.0 %)	141.36 ± 4.39 (43.36 %)	560.93 ± 18.19 (100.0 %)	268.06 ± 8.40 (41.87 %)	822.85 ± 55.61 (100.0 %)	396.77 ± 24.47 (42.86 %)	428.84 ± 28.77 (100.0 %)	192.94 ± 11.75 (44.58 %)	151.17 ± 9.89 (100.0 %)	82.31 ± 5.18 (35.88 %)	282.17 ± 19.43 (100.0 %)	144.98 ± 8.84 (43.06 %)	580.01 ± 38.66 (100.0 %)	274.86 ± 16.94 (41.53 %)
Fruit $mean ± sd (% total basket price)	107.78 ± 17.70 (13.55 %)	71.65 ± 6.40 (8.00 %)	55.32 ± 9.08 (13.35 %)	38.79 ± 3.48 (9.26 %)	19.08 ± 3.13 (13.03 %)	11.41 ± 1.02 (5.14 %)	38.15 ± 6.26 (13.98 %)	26.02 ± 2.31 (7.98 %)	74.39 ± 12.22 (13.26 %)	50.20 ± 4.50 (7.84 %)	119.20 ± 20.39 (14.49 %)	74.91 ± 8.86 (8.09 %)	61.18 ± 10.46 (14.27 %)	40.58 ± 4.86 (9.38 %)	21.10 ± 3.61 (13.96 %)	11.93 ± 1.43 (5.20 %)	42.19 ± 7.22 (14.95 %)	27.17 ± 3.14 (8.07 %)	82.28 ± 14.07 (14.19 %)	52.51 ± 6.30 (7.93 %)
Vegetables & legumes/beans $mean ± sd (% total basket)	131.65 ± 3.47 (16.55 %)	48.57 ± 0.50 (5.42 %)	66.29 ± 1.76 (15.99 %)	22.51 ± 0.21 (5.37 %)	25.88 ± 0.52 (17.67 %)	10.21 ± 0.13 (4.60 %)	42.76 ± 1.18 (15.67 %)	20.36 ± 0.27 (6.25 %)	92.17 ± 2.28 (16.43 %)	32.72 ± 0.33 (5.11 %)	134.82 ± 9.36 (16.38 %)	49.21 ± 1.98 (5.32 %)	67.81 ± 4.66 (15.81 %)	22.77 ± 0.78 (5.26 %)	26.43 ± 1.67 (17.49 %)	10.39 ± 0.52 (4.53 %)	43.87 ± 3.12 (15.55 %)	20.65 ± 0.98 (6.13 %)	94.25 ± 6.34 (16.25 %)	33.16 ± 1.31 (5.01 %)
Grains (cereal) foods $mean ± sd (% total basket price)	132.07 ± 0.24 (16.60 %)	67.29 ± 1.83 (7.51 %)	76.10 ± 0.14 (18.36 %)	32.97 ± 0.85 (7.87 %)	25.67 ± 0.05 (17.53 %)	13.050.43 (5.88 %)	32.12 ± 0.06 (11.77 %)	22.54 ± 0.65 (6.91 %)	101.78 ± 0.19 (18.14 %)	46.02 ± 1.28 (7.19 %)	136.17 ± 6.99 (16.55 %)	68.14 ± 2.86 (7.36 %)	78.46 ± 4.01 (18.30 %)	33.39 ± 1.37 (7.72 %)	26.47 ± 1.36 (17.51 %)	13.20 ± 0.61 (5.75 %)	33.13 ± 1.72 (11.74 %)	22.82 ± 0.98 (6.78 %)	104.93 ± 5.37 (18.09 %)	46.59 ± 1.97 (7.04 %)
Lean meats and poultry, fish eggs, tofu, nuts, seeds, legumes/beans $mean ± sd (% total basket price)	252.02 ± 8.73 (31.68 %)	143.48 ± 6.80 (16.01 %)	133.76 ± 5.00 (32.27 %)	66.53 ± 3.04 (15.89 %)	48.64 ± 1.40 (33.21 %)	35.63 ± 1.78 (16.06 %)	82.72 ± 2.56 (30.32 %)	55.96 ± 2.57 (17.16 %)	182.39 ± 6.38 (32.52 %)	101.77 ± 4.80 (15.90 %)	257.40 ± 11.62 (31.28 %)	147.08 ± 8.27 (15.89 %)	136.76 ± 6.27 (31.89 %)	68.04 ± 3.46 (15.72 %)	49.45 ± 2.15 (32.71 %)	36.59 ± 2.21 (15.95 %)	84.38 ± 3.72 (29.90 %)	57.42 ± 3.08 (17.06 %)	186.21 ± 8.43 (32.11 %)	104.24 ± 5.68 (15.75 %)
Milk, yoghurt, cheese and/or alternatives $mean ± sd (% total basket price)	160.00 ± 3.33 (20.54 %)	54.39 ± 0.98 (6.07 %)	79.42 ± 1.62 (19.16 %)	26.75 ± 0.49 (6.39 %)	24.20 ± 0.50 (16.53 %)	9.62 ± 0.16 (4.34 %)	74.15 ± 1.51 (27.17 %)	15.648 ± 0.28 (4.80 %)	103.62 ± 2.11 (18.47 %)	36.37 ± 0.65 (5.68 %)	166.35 ± 10.84 (20.22 %)	55.80 ± 3.61 (6.03 %)	80.88 ± 5.28 (18.86 %)	27.47 ± 1.83 (6.35 %)	24.64 ± 1.60 (16.30 %)	9.86 ± 0.60 (4.30 %)	75.52 ± 4.95 (26.76 %)	16.03 ± 1.01 (4.76 %)	105.52 ± 6.88 (18.19 %)	37.33 ± 2.43 (5.64 %)
Unsaturated Oils & Spreads $mean ± sd (% total basket price)	8.59 ± 0.28 (1.08 %)	1.57 ± 0.04 (0.18 %)	3.61 ± 0.12 (0.87 %)	0.65 ± 0.02 (0.16 %)	2.97 ± 0.10 (2.03 %)	0.33 ± 0.01 (0.15 %)	2.97 ± 0.10 (1.09 %)	0.85 ± 0.02 (0.26 %)	6.58 ± 0.22 (1.17 %)	0.98 ± 0.03 (0.15 %)	8.91 ± 0.83 (1.08 %)	1.64 ± 0.16 (0.18 %)	3.74 ± 0.35 (0.87 %)	0.68 ± 0.07 (0.16 %)	3.08 ± 0.29 (2.03 %)	0.34 ± 0.03 (0.15 %)	3.08 ± 0.29 (1.09 %)	0.89 ± 0.09 (0.26 %)	6.82 ± 0.63 (1.18 %)	1.02 ± 0.10 (0.15 %)
All discretionary choices $mean ± sd (% total basket price)		509.08 ± 17.16 (56.81 %)		230.51 ± 7.36 (55.05 %)		141.58 ± 3.76 (63.82 %)		184.63 ± 6.81 (56.64 %)		372.13 ± 11.04 (58.13 %)		528.93 ± 21.85 (57.14 %)		239.81 ± 9.69 (55.42 %)		147.12 ± 5.23 (64.12 %)		191.70 ± 8.05 (56.94 %)		387.05 ± 14.92 (58.47 %)
Alcoholic drinks $mean ± sd (% total basket price)		117.89 ± 0.62 (13.16 %)		40.72 ± 0.22 (9.73 %)		49.61 ± 0.38 (22.36 %)		66.43 ± 0.65 (20.38 %)		90.33 ± 0.59 (14.11 %)		116.92 ± 0.12 (12.63 %)		40.41 ± 0.03 (9.34 %)		49.23 ± 0.21 (21.46 %)		65.76 ± 0.63 (19.53 %)		89.64 ± 0.24 (13.54 %)
Take-away foods $mean ± sd (% total basket price)		123.63 ± 0.00 (13.80 %)		62.19 ± 0.00 (14.85 %)		31.53 ± 0.00 (14.21 %)		31.59 ± 0.00 (9.69 %)		93.49 ± 0.00 (14.60 %)		135.35 ± 0.00 (14.62 %)		68.14 ± 0.00 (15.74 %)		34.42 ± 0.00 (15.00 %)		35.10 ± 0.00 (10.42 %)		102.28 ± 0.00 (15.45 %)
Sugar-sweetened beverages $mean ± sd (% total basket price)		31.52 ± 0.00 (3.52 %)		16.67 ± 0.00 (3.98 %)		8.99 ± 0.00 (4.05 %)		5.11 ± 0.00 (1.57 %)		25.65 ± 0.00 (4.01 %)		32.01 ± 0.85 (3.46 %)		16.93 ± 0.45 (3.91 %)		9.13 ± 0.24 (3.98 %)		5.19 ± 0.14 (1.54 %)		26.04 ± 0.69 (3.93 %)

^a^ Results for current (unhealthy) diet in HH4 should be applied with caution

In this pilot study, the diet basket tools were developed when neither detailed confidential unit record data (CURFS) from the AHS 2011-12 [3] nor the food composition database (AUSNUT 2011–13) used in the analysis of that survey, were available publically. Although the draft tools appeared fit for purpose and performed well at face value at household level, differences between analysis of reported and constructed current diets at the individual level suggest that more accurate and specific current (unhealthy) diet basket tools may be able to be produced for Australia by accessing and analysing the AHS 2011–12 CURFS when available [3]. More broadly, it is noted that this more precise approach may be too complex for application globally, as detailed dietary survey data are not easily accessible in all countries and technical capacity to analyse individual records may be limited. Therefore the more pragmatic approach used in this pilot study may be more feasible for INFORMAS to monitor the price differential of healthy and current (unhealthy) diets globally [7].

### Diet prices

The price of the healthy (recommended) and current (unhealthy) diet baskets for each of the five household structures at the two locations is shown in Table 5. The higher variance at the high SES location was mainly due to the higher prices in the IGA store compared to the two major supermarket chains in that area. On average, food item prices tended to be around 3 % higher in the high SES location than the low SES location (Z = 2.49 *p* = 0.01). Take-away foods were relatively more expensive (approximately 9 %) in the high SES location than the low SES location (Z = 4.50, *p* < 0.01), but alcoholic drinks and sugar-sweetened beverages were priced similarly in both locations (Table 5).

All five household structures spend more purchasing current (unhealthy) diets than the amount required to purchase healthy (recommended) diets (Table 5). At both locations, a healthy diet cost 88–99 % of the money currently being spent on food and drinks in households containing children (HH1, HH2 and HH5). Relatively, a healthy diet costs even less than the current (unhealthy) diet in households without children; 84 % in the household of two elderly adults (HH4) and only 66 % in the household consisting of a single male (HH3).

Only 10–15 % of the food dollar is currently being spent on fruit and vegetables, compared to approximately 29 % required to achieve recommended intakes of these important foods. Similarly, less than half of the required amount is being spent on: grain (cereal) foods, particularly wholegrains (6–8 % currently compared to 11–18 % required); lean meats, poultry, fish, eggs and plant-based alternatives (16–17 % currently compared to 30–33 % required); milk, cheese, yoghurt (4–6 % currently compared to 17–27 % required); and unsaturated oils and spreads (<0.3 % currently compared to 1–2 % required).

Worryingly, the majority of the food dollar is being spent on discretionary choices at 53–64 % in both high and low socio-economic locations (Table 5). Within this category, 10–22 % of the total food dollar is spent on alcoholic drinks; this proportion is lowest in households containing children. If alcoholic drinks are excluded from the current diet, the cost of the healthy (recommended) diets is between 2–8 % more expensive than the current diet for all households except that of the single male (HH3). At 10–16 % of the current food dollar, spending is also relatively high on take-away foods (such as hamburgers and pizzas) and on sugar-sweetened beverages (1.6-4 %); spending for both these items is highest in families with children.

### Testing the impact of policy change on diet prices

In one example of policy change tested, proposal to expand the base of the GST to include fresh healthy food in Australia, the cost of the healthy (recommended) diet basket would significantly increase by 9.9 % but the cost of the current (unhealthy) diet basket would increase by only 4.5 % to 5.5 % (Z = −4.78, *P* < 0.01) (Table 6). In real terms, in order to purchase a healthy diet, on average the various households would need to find additional funds of: $80.15 for HH1; $41.86 for HH2; $14.52 for HH3; $27.40 for HH4; and $56.39 for HH5, whereas the additional cost of current (unhealthy) diets would increase by less than 45 % of these amounts. Under this scenario, healthy diets would become relatively more expensive than current (unhealthy) diets, and the discretionary items in the current (unhealthy diet) would become relatively more affordable also.

**Table 6:** Affordability of healthy (recommended) and current (unhealthy) diet baskets for five household structures for present pricing and projected pricing after potential changes to the Australian taxation system

	Logan										Indooroopilly									
Household	HH1		HH2		HH3		HH4^a^		HH5		HH1		HH2		HH3		HH4^a^		HH5	
HH income^b^ ($)	2679.38		1545.63		611.76		1389.40		2137.32		2679.38		1545.63		611.76		1389.40		2137.32	
Diet	Healthy	Current unhealthy	Healthy	Current unhealthy	Healthy	Current unhealthy	Healthy	Current unhealthy	Healthy	Current unhealthy	Healthy	Current unhealthy	Healthy	Current unhealthy	Healthy	Current unhealthy	Healthy	Current unhealthy	Healthy	Current unhealthy
Current Taxation system (Basic healthy foods exempt from GST)																				
Cost of diet/fortnight ($)	795.47	896.04	414.50	418.71	146.43	221.83	272.88	325.99	560.93	640.20	822.85	925.69	428.84	432.75	151.17	229.44	282.17	336.68	580.01	661.92
Affordability of diet (%)	29.69	33.44	26.82	27.09	23.94	36.26	19.64	23.46	26.24	29.95	30.71	34.55	27.75	28.00	24.71	37.50	20.31	24.23	27.13	30.97
GST base expanded to include basic healthy foods (10 %)																				
Cost of diet/fortnight ($)	874.17	942.27	455.63	440.78	160.70	231.72	299.80	343.54	616.33	672.13	904.36	974.29	471.43	455.84	165.95	239.95	310.05	355.20	637.38	695.49
Affordability of diet (%)	32.63	35.17	29.47	28.96	26.27	37.88	21.58	24.73	28.84	31.44	33.75	36.36	30.50	29.49	27.13	39.22	22.31	25.56	29.82	32.54
Extra total cost/ fortnight $ (%)	78.70 (9.89)	46.23 (5.16)	41.13 (9.92)	22.07 (5.27)	14.27 (9.75)	9.89 (4.46)	26.92 (9.87)	17.55 (5.38)	55.40 (9.88)	31.93 (4.99)	81.51 (9.90)	48.60 (5.25)	42.59 (9.93)	23.09 (5.34)	14.78 (9.78)	10.51 (4.58)	27.88 (9.88)	18.52 (5.50)	57.37 (9.89)	33.57 (5.07)
Extra cost of core foods /fortnight $ (%)	78.70 (9.89)	38.70 (10.00)	41.13 (9.92)	18.82 (10.00)	14.27 (9.75)	8.02 (10.00)	26.92 (9.87)	14.14 (10.00)	55.40 (9.88)	26.81 (10.00)	81.51 (9.90)	39.67 (10.00)	42.59 (9.93)	19.29 (10.00)	14.78 (9.78)	8.23 (10.00)	27.88 (9.88)	14.50 (10.00)	57.37 (9.89)	27.49 (10.00)
Extra cost of discretionary foods /fortnight $ (%)	0	7.54 (1.48)	0	3.25 (1.41)	0	1.87 (1.32)	0	3.40 (1.84)	0	5.13 (1.38)	0	8.92 (1.69)	0	3.80 (1.58)	0	2.28 (1.55)	0	4.02 (2.10)	0	6.09 (1.57)
Basic healthy foods exempt from GST but GST rate increased to 15 %^c^																				
Cost of diet/fortnight ($)	795.85	915.11	414.64	427.36	146.60	227.31	273.05	332.69	561.24	654.33	823.20	945.02	428.97	441.56	151.32	234.99	282.33	343.42	580.29	676.28
Affordability of diet (%)	29.70	34.15	26.81	27.63	23.96	37.16	19.65	23.94	26.25	30.61	30.72	35.27	27.74	28.56	24.74	38.41	20.32	24.72	27.15	31.64
Extra total cost increase/fortnight if GST increased to 15 % $ (%)	0.38 (0.05 %)	19.07 (2.13 %)	0.14 (0.03 %)	8.65 (2.07 %)	0.17 (0.12 %)	5.48 (2.47 %)	0.17 (0.06 %)	6.70 (2.06 %)	0.31 (0.06 %)	14.13 (2.21 %)	0.35 (0.04 %)	19.33 (2.09 %)	0.13 (0.03 %)	8.81 (2.04 %)	0.15 (0.10 %)	5.55 (2.42 %)	0.16 (0.06 %)	6.74 (2.00 %)	0.28 (0.05 %)	14.36 (2.17 %)
Basic healthy foods exempt from GST but GST rate increased to 20 %^c^																				
Cost of diet/fortnight ($)	796.24	934.82	414.78	436.36	146.76	232.90	273.21	339.54	561.55	668.91	823.56	965.01	429.10	450.74	151.48	240.64	282.48	350.31	580.58	691.10
Affordability of diet (%)	29.72	34.89	26.84	28.23	23.99	38.07	19..66	24.43	26.27	31.30	30.74	36.02	27.76	29.16	24.76	39.34	20.33	25..21	27.16	32.33
Extra total cost increase/fortnight if GST increased to 20 % $ (%)	0.77 (0.10 %)	38.78 (4.33 %)	0.28 (0.07 %)	17.65 (4.22 %)	0.33 (0.23 %)	11.07 (4.99 %)	0.33 (0.12 %)	13.55 (4.16 %)	0.62 (0.11 %)	28.71 (4.48 %)	0.71 (0.09 %)	39.32 (4.25 %)	0.26 (0.06 %)	17.99 (4.16 %)	0.31 (0.21 %)	11.20 (4.88 %)	0.31 (0.11 %)	13.63 (4.05 %)	0.57 (0.10 %)	29.18 (4.41 %)

^a^ Results for current (unhealthy) diet in HH4 should be applied with caution

^b^ Calculated low household income per fortnight. Median income per fortnight is $2,740 in Logan and $4,342 in Indooroopilly

^c^ All increased costs under this scenario pertain to discretionary food

If the GST base remained the same but the rate of GST was increased to 15 % or 20 % respectively, the price of the healthy diet basket would be similar to present, but the prices of the current (unhealthy) diet basket would increase by around 2 % and 4–5 % respectively, with the discretionary items in the current basket increasing by about twice these rates. Under the latter scenario, healthy diets would become relatively less expensive than current (unhealthy) diets.

### Affordability

The median family income per fortnight in 2011 was $4,342 in the high SES area and $2740 per fortnight in the low SES area [28]. In the high SES area, the cost of the healthy and current (unhealthy) diet baskets for the average family household per fortnight is $610.56 (14 % of median income) and $673.45 (15.5 % of median income) respectively. In the low SES area, the cost of the healthy and current (unhealthy) diet baskets for the average family household per fortnight is $590 [28] (21.5 % of median income) and $651.65 (23.7 % of median income) respectively.

The affordability of the diet baskets in low income households is nearly half that of families of median income (Table 6). Affordability of the healthy diet basket as a proportion of household income ranges from around 20 % for the low income household of two pensioners (HH4) to around 30 % for the low income household of six (HH1).

However, for these low income families, under the mooted changes to expand the base of the GST to include basic healthy food in Australia, the affordability of the healthy (recommended) diet would decrease by around 4 %, but the affordability of their current (unhealthy) diet would decrease by only around 2 % (Table 6). However, if the GST exemption on basic healthy foods was retained, but the rate on other foods and goods and services was increased by 15 % or 20 %,a healthy diet would be 7 % and 9 % respectively more relatively affordable than an unhealthy diet (Table 6); but the pressure on the food budget would likely be increased due to the increased cost of other essential items [29].

## Discussion

### Price and price differential of healthy (recommended) and current (unhealthy) diets

This study aimed to pilot the development and testing of draft tools, survey protocols, data collection and analysis systems to investigate the price, relative price and affordability of healthy (recommended) and current (unhealthy) diets in Australia, as well as test the utility of the approach to assess impacts of potential policy changes on diet prices, in this case the proposed broadening and/or raising of the GST base in Australia.

Findings suggest that healthy diets consistent with national dietary recommendations are less expensive than current (unhealthy) diets consumed by the Australian population; however lower income households are still required to spend a high proportion of their disposable income accessing healthy foods. As there is a common perception that healthy foods are more expensive than unhealthy foods [7, 31, 32] these results may be considered surprising. A systematic review that accounted for key sources of heterogeneity, found little difference between the prices of healthier versus less healthy dietary patterns, although the former tended to be slightly more expensive [31]. The effect of the cost of alcoholic drinks and pre-prepared ‘convenience’ foods do not appear to have been considered in that review, which may help explain why our findings differ from these results as the current (unhealthy) Australian diet includes a high proportion of these items. Amongst other components of the diet, depending on the unit reported (that is, per energy, weight or serving), the individual price of some healthy foods, particularly meats and dairy foods and vegetables, are relatively expensive compared with energy dense discretionary foods and are also more expensive than core (healthy) cereal-based foods [31, 33].

The high proportion of the food dollar currently being spent on discretionary choices in Australia is of particular concern. For example, a family of two adults and two children (HH5) spends over 58 % of their food dollar on discretionary choices, which provide around 38 % of their total energy intake. A recent report on the Australian Dietary Guidelines food price indexes released by the Australian Government [34] found that 58.2 % of household’s food budget in 2014 was spent on discretionary foods and drinks, confirming our findings. Within the discretionary category, we found households are spending around 14 % on alcoholic drinks, around 15 % on take-away foods and around 4 % on sugar-sweetened beverages. This suggests that, while food price is important, other factors such as convenience and/or desirability and ‘taste’, and the determinants of these factors, such as the ubiquitous availability, advertising and marketing of discretionary choices, poor food literacy and cooking skills and busy lifestyles may be more important influences on food choice in Australia [4].

Higher food prices were observed in the higher SES area compared to the lower SES area consistent with previous studies [35].

No attempt was made to control the price of the healthy (recommended) diet baskets and the current (unhealthy) diet baskets for energy, as the diets are constructed on recommended energy levels and actual reported levels of energy respectively, and the energy content of each is a determinant variable that directly affects diet-related health outcomes [4, 7]. Further, as the key exposure variable affecting the life time risk of diet-related disease is the total diet and dietary patterns, these findings illustrate that studies such as this pilot that compare the cost of actual diets with recommended diets are more pertinent to the health policy debate than the more common, but limited, studies into the relative price of selected ‘healthy’ and ‘unhealthy’ foods or single ‘optimised’ diets [7, 18, 19, 31].

### Affordability of healthy (recommended) and current (unhealthy) diets

Findings suggest that a healthy diet consistent with Australian Dietary Guidelines [4] is presently affordable for families on a median income costing approximately 18 % of disposable income, but is much less affordable for low income families, costing around 28 % of their household disposable income (Table 6). In comparison, current (unhealthy) diets cost around 20 % of the income of a family of median income and 32 % of the income of low income families (Table 6).

These results are consistent with household expenditure survey data [30] which showed that 16.5 % of average equivalised disposable income was spent on food and drink in all Queensland households, but at 21–41 % of disposable income of low income households, are higher than the equivalised proportion (19.5 %) reported for these households in another study [29]. Disparities are likely to be due to different methodologies, but the amounts spent on food and drink per week in the official reports [29, 30] (for example $79 per week for an unemployed single person) are very low suggesting very restricted diets, and do not concord with prices of the mean quantities of food and drink reported in the AHS [3].

Our findings are similar to those community studies of the affordability of selected ‘healthier’ diets (i.e., not necessarily consistent with dietary recommendations) which cost between 28 % and 40 % of the disposable income of lowest income families, compared with 20 % for families on the average income [7] and up to 48 % of income if environmental sustainability of the diet is also considered [5]. In Aboriginal and Torres Strait Islander communities in Australia, depending on location, it has been estimated that at least 34 %-80 % of the family income is needed to purchase healthy diets [9, 36]. At least 3.7 % of Australian households report having run out of food in the previous 12 months and not being able to afford to buy more [3, 6]. This proportion is higher among some groups, affecting more than one in in five Aboriginal and Torres Strait Islanders (22 %) [37], around 11 % of those unemployed and 16 % of rental households [6].

Internationally there is no accepted benchmark for affordability of a healthy diet [7]. One of the proposed equity targets to close the gap on Indigenous disadvantage in Australia was that, by 2018, 90 % of Indigenous families could access a healthy food basket for under 25 % of their disposable income [38]. A figure of 25 % seems reasonable as a working estimate given the competing priorities for other essential items in the household budget, such as housing, transport, health services, clothing and utilities [29].

The fact that current (unhealthy) diets including alcoholic drinks cost more than healthy diets in Australia, may mask the fact that, at 28 % of income, healthy diets are already unaffordable for low income families in Australia, and reinforces the notion that, if we are not to worsen diets and increase the prevalence of diet-related chronic disease in Australia, price-elasticities must be considered very carefully before food prices are changed in Australia, as proposed under potential changes to the Australian Tax System [20]. Quantitative modelling of the impacts of such strategies requires an understanding of own-price elasticities (how consumption changes with an item’s own price) and cross-price elasticities (how consumption of an item changes with changes in the price of another good) in different groups [39, 40]. An additional consideration for food price elasticities is whether the cross-price elasticities are within the same or different food groups [18]. Plausible food price elasticities have been estimated for use in Australia and New Zealand [40] and globally [41] and could be used to model the consumption and consequent health impacts of the changing prices estimated under various policy scenarios in future studies.

The poor quality of the current Australian diet is consistent with high risk of diet-related disease, especially those mediated by obesity, including type 2 diabetes, cardiovascular disease and some cancers [4] and represents a significant threat to Australia’s health, economic and broader social systems [1, 4]. Therefore it is critical that no additional barriers, such as increased cost of healthy foods, are put in the way of Australians choosing healthier foods and diets.

The findings suggest that more needs to be done to promote healthy diets, including disseminating the fact that they can be less expensive than current dietary patterns. Healthy budgeting programs such as *FOODcents* may be useful in this regard [42]. Affordability of healthy foods among lower income groups could be improved by promoting cheaper options that are still healthy, such as local specials and seasonal or irregular shaped fresh produce. It is also likely that barriers to healthy choices, such as low food literacy and poor cooking skills, also need to be addressed [4, 43]. However, there is mounting evidence that current “food environments exploit people’s biological, psychological, social and economic vulnerabilities, making it easier for them to eat unhealthy foods” [44] and that “regulatory actions from governments and increased efforts from industry and civil society will be necessary to break these vicious cycles” [44].

### Testing impact of policy changes on diet prices: the effect of potential changes to the Australian taxation system

Under potential changes to the Australian Tax System [20], if the GST base was extended to encompass basic healthy foods, the cost of healthy (recommended) diets would increase (9.9 %) by more than double the rate of current (unhealthy) diets (4.5 %), decreasing the relative affordability of healthy foods. In real terms, a healthy diet would cost a family of two adults and two children an additional $56.39 per week. At 30 % of disposable household income, this is likely to be unaffordable for low income families. Under this scenario, it would be less likely that the population would move towards healthier eating patterns, as the healthy foods consumed within the current diet would also increase significantly in price compared with discretionary choices; hence it would be likely that an even greater proportion of the food dollar would be spent on discretionary choices (‘junk food’).

If the GST is not extended to basic healthy foods but is applied at higher rates (15 % and 20 %) to those foods and drinks currently incurring GST, a healthy diet would cost no more than presently, but the cost of the current (unhealthy) diet would increase by more than $14.00 (2.2 %) and around $29.00 (4.5 %) per fortnight respectively for a family of two adults and two children. As the relative affordability of a healthy diet compared with the current (unhealthy) diet would improve under these scenarios, these policy actions effectively work as a tax on unhealthy food [19]. However the cost of non-food items that currently attract GST at 10 % would also increase, potentially leaving less money to be spent on food, highlighting the regressive nature of GST which disproportionally affects those on lower incomes [19]. Consideration of available price elasticities in Australia [40] and recent ‘real world’ data showing that a 20 % price reduction in fruit and vegetables increased household purchases by 35 % for fruit and 15 % for vegetables [45] suggests that the higher rate of GST would be most effective in helping drive dietary improvements. However, as mentioned previously, careful consideration of factors other than price that influence dietary choices would also be required. In addition, a healthy diet would still cost up to 28 % of the disposable income of low income families and would be unaffordable for some groups. The regressive nature of taxation on unhealthy foods is potentially problematic but can be alleviated by targeted subsidies to vulnerable groups to help alleviate pressure on the household budget [7, 19]. Differential tax rates, such as 30 % GST on sugar-sweetened beverages, may also have merit [19].

### Limitations

There are several methodological limitations in this study, including that the cost of the current (unhealthy) diet may not be the same as actual expenditure on food, given that it is based on national mean dietary intakes. Other assumptions include that food is shared equitably throughout the households, that there is no home food production and no wastage. Nutritionally similar products were aggregated to minimize the number of items included in the food pricing basket tools but products were not necessarily homogenous in terms of price; similar food items were included in each basket to try to minimize any unintended effects. No adjustments were made for costs such as transport, time, cooking equipment and utilities; as these apply to both the current and healthy (recommended) diets, assessment of the price differential between the two can control for some of these hidden costs to some extent, but the effect of these would increase actual diet costs and decrease affordability of the diets. No adjustments were made to account for the marked under-reporting in the AHS 2011–12 [3, 37], reported dietary variability amongst different groups other than age/gender stratification, the greater proportion of pre-prepared ‘convenience’ items containing meats and/or vegetables the current (unhealthy) diet tool, or, given the high rates of overweight/obesity in Australia, that the Foundation diets were prescribed for the shortest and least active in each age group [17]. The study design presumes that price and healthfulness are key drivers of dietary choice in families, but as the results suggest, other needs such as taste and convenience, may now play a greater role in food choice.

Arbitrary decision points occur around sampling frameworks, data collection protocols, analysis and presentation of results, data sources and definitions of family and household income and composition. Such methodological limitations are common to other food price studies, and in order for final methods to be replicable, detailed publication of all assumptions and protocols, and the underlying rationale of the final methods is required.

### Next steps

Our next step is to apply insights from this pilot study to finalize the diet basket pricing tools. We are reconstructing the composition of the current (unhealthy) baskets using detailed unit record data (CURFS) from the AHS 2011–12 [3], analyzing these with the AUSNUT 2011–13 food composition data base and documenting key decision points in the survey protocols to produce a detailed methods paper for the final tools.

Once the tools are finalized, a workshop with all stakeholders will be held to build on previous understandings and seek agreement for the use of these methods as a standardized approach throughout Australia. Pending funding, additional data will be collected in New South Wales, the Australian Capital Territory, Queensland and, ideally, nationally as part of the first INFORMAS assessment of Australian food environments.

Resultant food price and affordability data will be used to feed into broader computer modelling systems, for example, to determine more robust estimates of projected health impacts and costs under different fiscal policy scenarios.

## Conclusion

This pilot study confirms that, to inform fiscal and health policy actions and ensure equitable affordability of healthy diets, standardized food price assessment, monitoring and surveillance efforts should seek to determine and compare the costs of current (unhealthy) diets as well as healthy (recommended) diets. Our findings suggest that the approach suggested in this paper is a valid means of achieving this.

The results show that Australians are spending more on current (unhealthy) diets than would be required to purchase healthy diets consistent with the recommendations of the Australian Dietary Guidelines. Using the methods to test the price impacts of policy options, showed that proposed extension to the GST base to include basic healthy food would markedly increase the cost of a healthy diet relative to current (unhealthy) diets, limiting the affordability of healthy dietary patterns, particularly for lower income households who are already most vulnerable to poor diet-related health.

Findings also suggest that more needs to be done to tackle assumptions that healthy diets are more expensive than unhealthy diets. More research is required to better understand the relative importance of factors affecting food choice in Australia.

The study has demonstrated that meaningful food pricing tools based on recent Australian dietary data (current unhealthy diet) and recommendations (healthy diet) can be drafted and applied readily. The draft tools appeared fit for purpose and performed well at face value at household level, but some improvements can and will be made. The tools have the potential to be standardized nationally and inform similar research in other countries.

### Ethics and consent to participate

The QUT University Human Research Ethics Committee assessed this study as meeting the conditions for exemption from Human Research Ethics Committee review and approval in accordance with section 5.1.22 of the National Statement on Ethical Conduct in Human Research (2007); the exemption number is 1500000161. All data were obtained from publically available sources and did not involve human participants, so consent to participate is not applicable.

### Consent to publish statement

Not applicable.

### Availability of data and materials

Data sets have been included as Additional file 1.

## Additional file

Additional file 1: Detailed calculation of diet basket contents and price. (XLSX 485 kb)

## Abbreviations

ABS: Australian Bureau of Statistics
AHS: Australian Health Survey
CURFS: Confidentialised Unit Record Files
GST: Goods and Services Tax
IGA: Independent Grocers Australia
INFORMAS: International Network for Food and Obesity/non-communicable diseases Research Monitoring and Action Support
NCDs: non-communicable diseases
NUTTAB: Nutrient Tables for use in Australia
PAL: physical activity level
QCOSS: Queensland council of social services
SA2: statistical area level 2
SES: socio-economic status
